# Association of left ventricular mass with discordant stress cardiac magnetic resonance and coronary angiography

**DOI:** 10.1093/ehjci/jeaf350

**Published:** 2026-02-10

**Authors:** Kanyaw Kader, Laust Dupont Rasmussen, Salma Raghad Karim, Jelmer Westra, Christin Isaksen, Jacob Hartmann Søby, Jonathan Nørtoft Dahl, Lau Brix, Steffen E Petersen, Theodore Murphy, Simon Winther, Evald Høj Christiansen, Morten Böttcher, Ashkan Eftekhari

**Affiliations:** Department of Cardiology, Aalborg University Hospital, Aalborg, Denmark; Department of Cardiology, Aalborg University Hospital, Aalborg, Denmark; Department of Cardiology, Gødstrup Hospital, Herning, Denmark; Department of Cardiology, Aarhus University Hospital, Aarhus, Denmark; Department of Cardiology, Aarhus University Hospital, Aarhus, Denmark; Department of Cardiology, Linköping University Hospital, Linköping, Sweden; Department of Radiology, Regional Hospital Silkeborg, Aarhus, Denmark; Department of Cardiology, Gødstrup Hospital, Herning, Denmark; Department of Cardiology, Gødstrup Hospital, Herning, Denmark; Department of Radiology, Regional Hospital Silkeborg, Aarhus, Denmark; Comparative Medicine Lab, Department of Clinical Medicine, Aarhus University, Aarhus, Denmark; Barts Heart Centre, St Bartholomew’s Hospital, Barts Health NHS Trust, West Smithfield, London, United Kingdom; William Harvey Research Institute, NIHR Barts Biomedical Research Centre, Queen Mary University London, Charterhouse Square, London, United Kingdom; Barts Heart Centre, St Bartholomew’s Hospital, Barts Health NHS Trust, West Smithfield, London, United Kingdom; Department of Cardiology, Gødstrup Hospital, Herning, Denmark; Department of Cardiology, Aarhus University Hospital, Aarhus, Denmark; Department of Cardiology, Gødstrup Hospital, Herning, Denmark; Department of Clinical Medicine, Aarhus University, Aarhus, Denmark; Department of Cardiology, Aalborg University Hospital, Aalborg, Denmark

## Abstract

**Aims:**

This study aimed to determine the impact of left ventricular mass (LVM) on discordant stress cardiac magnetic resonance (CMR) imaging and invasive coronary angiography (ICA) in patients with suspected coronary artery disease (CAD) at coronary computed tomography angiography (CCTA).

**Methods and results:**

In this substudy of the Dan-NICAD 2 trial (NCT03481712), 354 patients with suspected obstructive CAD on CCTA were examined with both rest and stress CMR and ICA for invasive physiological measurements. An abnormal stress CMR was defined as ≥2 contiguous segments with a stress perfusion defect, late gadolinium enhancement, or wall motion abnormality. CMR-derived LVM was sex-adjusted by conversion from grams to per cent. Haemodynamically obstructive CAD at ICA was defined as visual diameter stenosis >90% or FFR ≤0.80. LVM was higher in patients with an abnormal stress CMR compared to those with a normal CMR (median difference = 8.0%, *P* < 0.001). Patients with or without haemodynamically obstructive CAD had similar LVM (median difference = 2%, *P* = 0.222). Within four binary groups based on normal/abnormal stress CMR and ICA, both median LVM and index of microvascular resistance were higher in patients with discordant abnormal stress CMR and normal ICA than in patients with concordant normal stress CMR and ICA (124% vs. 111%, *P* = 0.001, and 29 vs. 19, *P* = 0.072, respectively).

**Conclusion:**

In patients with suspected obstructive CAD, increased LVM can potentially confound concordance between stress CMR and ICA. This is due to increased microvascular resistance, which decreases the pressure gradient across an epicardial stenosis, resulting in a false high FFR and thus, normal ICA.

## Graphical Abstract

**Graphical Abstract:**
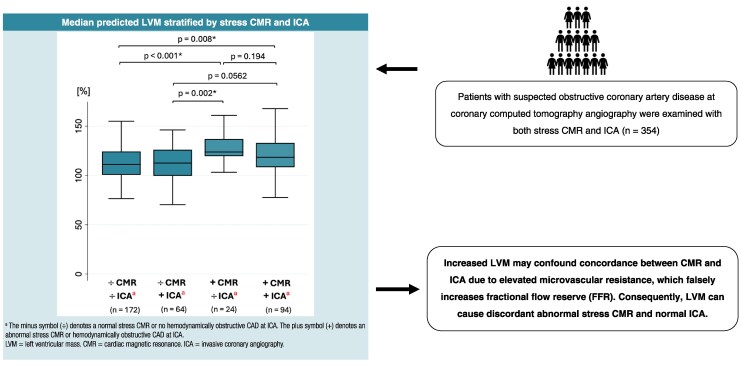
Study design and main results.

## Introduction

Coronary computed tomography angiography (CCTA) is recommended as the first-line non-invasive anatomical imaging in patients with low-intermediate pre-test likelihood of coronary artery disease (CAD) to rule out obstructive lesions.^1,2^ However, CCTA overestimates CAD severity, and one-in-two patients do not undergo revascularization after an invasive angiography with a preceding CCTA.^3^

Therefore, in patients with new-onset chest pain and clinically suspected obstructive CAD, guidelines recommend downstream non-invasive imaging before referral to invasive angiography to avoid unnecessary invasive coronary angiographies (ICA) and to verify inducible ischaemia following a CCTA-suspected stenosis.^1,2^ One method for this follow-up investigation is cardiac magnetic resonance (CMR) imaging, which has shown high diagnostic accuracy for obstructive CAD in cohorts with high-risk patients and high disease prevalence.^4^ However, guidelines recommend revascularization based on fractional flow reserve (FFR) ≤ 0.80.^5^ Furthermore, the ability of CMR to identify invasively assessed CAD is limited in patients with a preceding CCTA and a moderate pre-test likelihood of obstructive CAD.^6,7^

Previously, it has been proposed that increased left ventricular mass (LVM) is associated with myocardial ischaemia because of a decrease in coronary lumen volume to myocardial mass ratio, which, additionally, has been related to impaired invasive measures of FFR ≤0.80.^8^ However, the impact of LVM on discordant CMR and ICA findings regarding haemodynamically obstructive lesions has not been thoroughly addressed.

This study aimed to determine the impact of LVM on discordant stress CMR and ICA findings in patients with suspected CAD.

## Methods

### Study design and population

**Figure 1:**
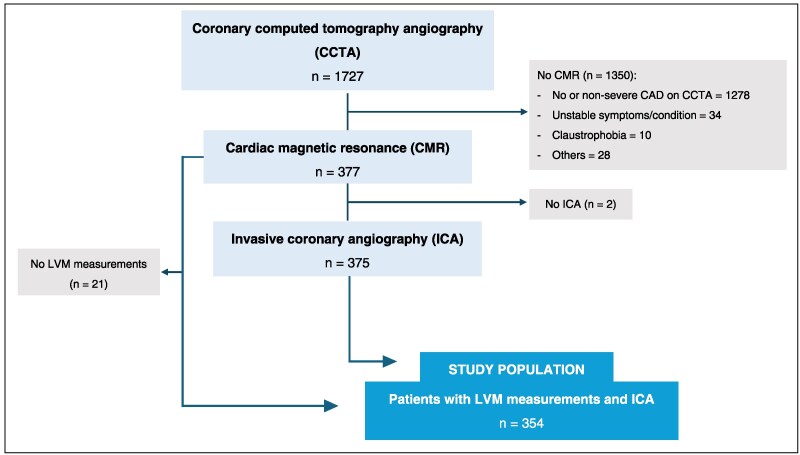
Flowchart of study design. CAD, coronary artery disease; LVM, left ventricular mass.

This was a substudy of the Danish study of Non-Invasive testing in Coronary Artery Disease (Dan-NICAD) 2 trial (NCT03481712). A thorough study protocol, including inclusion and exclusion criteria, has previously been published.^9^ In brief, this cross-sectional, multicentre study included 1734 patients with low to intermediate pre-test likelihood of obstructive CAD but without history of CAD. All patients underwent CCTA, and if CCTA suspected obstructive stenoses (diameter stenosis ≥50%), patients were further referred to myocardial perfusion imaging including 3.0T CMR, and subsequent ICA including measures of FFR (*Figure 1*). The ICA operator was blinded to the results of the myocardial perfusion imaging (MPI) modalities.

This study assessed the impact of LVM on discordant stress CMR and ICA findings of haemodynamical impairment using logistic regression analyses. Patients were stratified by CMR and ICA abnormality by pre-specified thresholds. Analyses included distributive LVM means across arbitrary groups of CMR findings and other invasive physiological measures, i.e. the coronary flow reserve (CFR) and the index of microvascular reserve (IMR). The primary analyses of the association of LVM with discordant CMR and ICA findings were conducted on a per-patient level.

### Imaging

#### Coronary computed tomography angiography

CCTA images were obtained using a 320 multi-slice volume computed tomography (CT) scanner (Aquilion One, Toshiba Medical Systems, Japan, and Siemens Flash, Siemens Healthcare, Germany) with prospective electrocardiogram triggering. All images were analysed onsite by experienced cardiologists in a dedicated workstation (Vitrea Advanced Workstation, Vital Images, MN, USA, or Syngo.Via, Siemens Healthcare, Erlangen, Germany). Suspected obstructive CAD was defined as segments with ≥50% diameter stenosis using the 18-segment model of the coronary tree.

#### CMR imaging

CMR scans were performed using a 3.0T MRI system (Siemens Skyra, Software release E11A, Siemens Healthcare GmbH, Germany) using the Body 18 and Spine 32 receive coils. Stress perfusion was performed upon intravenous adenosine infusion 140 µg/kg/min, followed by a rest perfusion examination after washout of the stress agent. Myocardial blood flow was assessed using injection of a gadolinium contrast agent (Gadovist®, Bayer Schering Pharma AG, Germany) during stress and rest, after which a late gadolinium enhancement (LGE) protocol was performed to assess cardiac viability. Abnormalities in ventricular wall motion were visually assessed from 1 to 5 (1 = normal, 2 = mild hypokinesia, 3 = severe hypokinesia, 4 = akinesia, and 5 = dyskinesia).

Analyses of CMR scans were conducted in an independent core laboratory (William Harvey Research Institute, Queen Mary University of London, London, UK), blinded to patient characteristics and results of previous examinations. An abnormal CMR was pre-defined as ≥ 2 contiguous segments with either a significant perfusion defect (either subendocardial or transmural signal changes), LGE, and/or wall motion abnormalities. For both LGE and wall motion assessment, the threshold for abnormality was solely based on ischaemic changes, ruling out inclusion of patients with non-ischaemic patterns.^7^

Left ventricular myocardial mass was assessed using a short-axis balanced steady-state free precession cine sequence. The endocardial and epicardial borders of the left ventricle in both end-diastolic and end-systolic phases were automatically segmented using CVI42 (Release 5.16, Circle Cardiovascular Imaging Inc., Calgary, Canada) software and could be manually corrected. The volumes were automatically calculated for end-diastolic volume and end-systolic volume phases. The myocardial mass was calculated using the formula:


$$m_{myo}=(V_{epi}-V_{endo})\times \;\rho_{tissue}$$

where *m*_myo_ is myocardial mass, *V*_epi_ and *V*_endo_ are epicardial and endocardial volumes, respectively, and *ρ*_tissue_ is the tissue density in g/cm^3^.

LVM was then sex-adjusted to allow comparison across sex by converting LVM from grams to per cent as suggested in The Multi-Ethnic Study of Atherosclerosis (MESA) trial^10^ (see Supplementary data online, *Equation S1*). In the trial, left ventricular hypertrophy (LVH) was defined as LVM greater than the 95th percentile (equivalent to >136%).

#### Invasive coronary angiography

Prior to ICA, 250 μg of intracoronary nitroglycerine and 5000 IU heparin were administered. All lesions with a diameter stenosis of 30–90% by visual assessment and a reference diameter of >2 mm were considered for physiological assessment in the main Dan-NICAD 2 study. The pressure-wire (PressureWire × Guidewire, Abbott, Chicago, IL) and CoroFlow (Coroventis Research AB, Uppsala, Sweden) systems were used according to manufacturer instructions.

Hyperaemia was induced using intravenous adenosine (140 µg/kg/min). Functional measurements of FFR were obtained during maximum hyperaemia when distal pressure (Pd)/aortic pressure (Pa) were stable. Routine checks were made to ensure that drift did not occur after the recordings, and an absolute drift value of ≤±0.02 was accepted. CFR and IMR were measured with three bolus injections of 3 mL saline to obtain hyperaemic thermodilution curves for mean transit time calculation during rest and maximum hyperaemia. CFR was calculated as the average hyperaemic mean transit time/resting mean transit time. IMR was calculated as hyperaemic transit time × Pd_(hyperaemia)_.

Three-dimensional quantitative coronary angiography (QCA) was performed with two angiograms with 15 frames/s without panning, no foreshortening, and at least 25° apart.

All physiological core laboratory analyses were performed blinded to the baseline characteristics and non-invasive imaging examinations. Invasive physiology traces were evaluated in an offline core laboratory (Institute of Clinical Medicine, Aarhus University, Denmark).

Haemodynamically obstructive CAD was pre-defined as a high-grade stenosis (diameter stenosis >90%) by visual assessment or FFR ≤0.80 in a vessel with a diameter stenosis of 30–90% or QCA based diameter stenosis (≥50% diameter) if FFR was indicated but not performed. For the analyses of the association between LVM, CFR, and IMR, cut-off values of CFR <2.5 and IMR >25 were defined as abnormal.^11^

### Statistical analysis

Categorical variables were expressed as numbers and percentages and were compared using *χ*^2^ test or Fisher’s exact test if the assumptions of a *χ*^2^ test were violated. Continuous variables were expressed with means and standard deviations, or median and interquartile range (IQR) if data did not follow normal distribution by Shapiro–Wilk test. Medians were compared using Mann–Whitney *U* test.

Univariate logistic regressions assessed the association between LVM and the probability of an abnormal CMR or heamodynamically obstructive CAD at ICA. This was followed by multivariate analyses to determine the impact of pre-defined risk factors of CAD on the regression model, namely age, sex, smoking, body mass index (BMI), hypertension, diabetes, FFR, CFR, and IMR. Results were presented with odds ratios (OR) and a 95% confidence interval (CI). For LVM, OR were presented per 5% increase in LVM. In both uni- and multivariate analyses, FFR, CFR, and IMR were converted to categorical variables based on pre-defined thresholds. This was done to only include physiological measurements in coronary lesions with a visual diameter stenosis 30–90%.

Patients were classified into four binary concordant or discordant groups according to CMR abnormality and the presence of haemodynamically obstructive CAD at ICA, respectively. In concordant outcomes, the result of CMR and ICA were both either normal or abnormal, whereas discordant outcomes warranted incongruent findings at CMR and ICA. A Kruskal–Wallis *H* test was performed to assess overall difference within the binary groups, and if significant, a *post hoc* Dunn’s test with Bonferroni correction was done.

For all analyses, a two-sided *P*-value <0.05 was considered statistically significant. Statistical analyses were conducted in Stata/MP 17.0 (StataCorp, College Station, TX, USA).

## Results

### Study characteristics

In total, 375 patients underwent both stress CMR imaging and ICA, of whom LVM measurements were assessed in 354 (94%) patients. Most patients were male (71%), and median age was 64 (58–70) years (*Table 1*). Hypertension was the predominant cardiovascular risk factor and was present in 180 (51%) patients.

**Table 1:** Subject and haemodynamic characteristics

	Study population (*n* = 354)
Sex	
Male	251 (71)
Age [years]	64 (58–70)
Risk factors	
Hypertension	180 (51)
Diabetes mellitus	34 (9.6)
Active smoking	118 (33)
Family history of CAD^a^	133 (38)
BMI	27 (25–29)
Symptoms	
Typical angina	102 (29)
Atypical angina	127 (36)
Non-specific chest pain	82 (23)
Others e.g. dyspnoea or arrhythmia	43 (12)
Invasive haemodynamic parameters in coronary lesions with DS 30–90%	
FFR (*n* = 158)	0.83 (0.75–0.89)
CFR (*n* = 166)	2.3 (1.7–3.1)
IMR (*n* = 165)	19 (14–28)

Values expressed as *n* (%) or median (IQR).

^a^Defined as a 1st degree relative with early signs of ischaemic heart disease (men <55 years old, women <65 years old).

CAD, coronary artery disease; BMI, body mass index; DS, diameter stenosis; FFR, fractional flow reserve; CFR, coronary flow reserve; IMRindex of microvascular reserve.

### The impact of LVM and invasive measures on CMR

**Figure 2:**
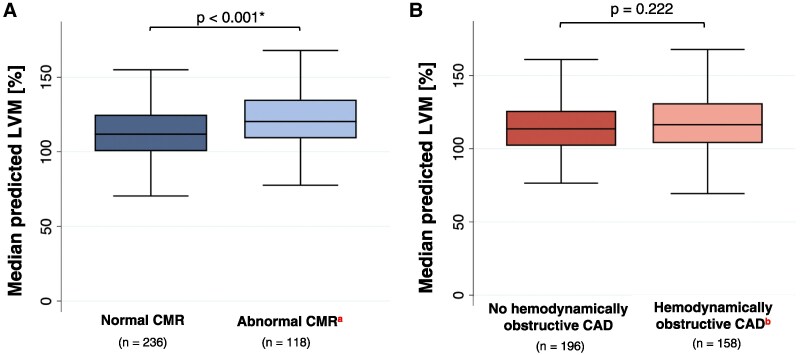
Median predicted LVM stratified by CMR and ICA. Boxplots displaying median (IQR) predicted LVM [%] stratified by *A*) stress CMR result and *B*) haemodynamically obstructive CAD. Difference in median LVM calculated by Mann–Whitney *U* test. Significant *P*-values marked with *. ^a^ Panel A: Defined as a significant perfusion defect in ≥2 contiguous segments and/or LGE in ≥2 contiguous segments and/or wall motion abnormalities in ≥2 contiguous segments. ^b^ Panel B: Defined as a visual diameter stenosis >90% or FFR ≤0.80 in lesions with 30–90% diameter stenosis during ICA. LVM = left ventricular mass; CMR, cardiac magnetic resonance; CAD, coronary artery disease.

Of the 354 patients, 118 (33%) had an abnormal CMR. With some overlap, 94/118 (80%) had stress perfusion defects, 19/118 (16%) had LGE, and 32/118 (27%) had stress-induced wall motion abnormalities. Median predicted LVM was higher in patients with an abnormal CMR compared to patients with a normal CMR [median predicted LVM = 120% (IQR: 109–135) vs. median predicted LVM = 112% (IQR: 100–125), respectively, *P* < 0.001] (*Figure 2A*). A total of 49/354 (14%) patients had LVH according to the above definition of >136%, of which 26 (53%) had an abnormal myocardial perfusion on CMR.

In both uni- and multivariate analyses, higher median predicted LVM was associated with an increased likelihood of an abnormal CMR; OR = 1.123, 95% CI: 1.062–1.202 (*P* < 0.001) and OR = 1.159, 95% CI: 1.013–1.326 (*P* = 0.032), respectively (*Table 2*). An FFR ≤0.80 in coronary lesions with a diameter stenosis of 30–90% was also associated with an abnormal CMR in both univariate (OR = 5.266, 95% CI: 2.286–12.131, *P* < 0.001) and multivariate analysis (OR = 4.542, 95% CI: 1.691–12.198, *P* = 0.003).

**Table 2:** Association of demographic variables and intracoronary measures at ICA on CMR outcome

	Univariate analysis		Multivariate analysis	
	OR (95% CI)	*P*-value	OR (95% CI)	*P*-value
Age	0.991 (0.964–0.1.020)	0.557	0.994 (0.937–1.055)^a^	0.855
Male	3.127 (1.772–5.518)	<0.001	1.394 (0.424–4.580)^a^	0.585
Hypertension	1.290 (0.828–2.011)	0.260	1.099 (0.428–2.821)^a^	0.844
Diabetes	1.268 (0.611–2.630)	0.524	2.024 (0.580–7.063)^a^	0.269
BMI	1.066 (1.007–1.128)	0.028	1.129 (0.990–1.1288)^a^	0.070
Active smoking	0.709 (0.427–1.175)	0.182	0.302 (0.887–1.025)^a^	0.055
Median predicted LVM^b^	1.123 (1.062–1.202)	<0.001	1.159 (1.013–1.326)^a^	0.032
FFR ≤ 0.80	5.266 (2.286–12.131)^a^	<0.001	4.542 (1.691–12.198)^a^	0.003
CFR < 2.5	0.734 (0.347–1.551)^a^	0.418	0.606 (0.237–1.544)^a^	0.294
IMR > 25	0.838 (0.359–1.955)^a^	0.683	1.223 (0.409–0.3.661)^a^	0.719

Univariate and multivariate regression analysis between dependent variable (CMR outcome) and independent variables (age, sex, hypertension, diabetes, BMI, active smoking, and LVM, FFR, CFR, and IMR). Data expressed with OR (95% CI).

^a^Analyses performed in patient with coronary lesions with visual DS 30–90% (*n* = 183)

^b^For every 5% increase in median predicted LVM.

CMR, cardiac magnetic resonance; OR, odds ratio; CI, confidence interval; BMI, body mass index; LVM, left ventricular mass; FFR, fractional flow reserve; CFR, coronary flow reserve; IMR, index of microvascular resistance.

### The impact of LVM and invasive measures on haemodynamically obstructive CAD

Haemodynamically obstructive CAD at ICA was found in 158/354 (45%) patients; 81/159 (51%) were based on a high-grade stenosis, 61/158 (39%) on FFR ≤0.80, and 16/158 (10%) on QCA ≥50% diameter stenosis.

Median predicted LVM was similar in patients with and without haemodynamically obstructive CAD [116% (IQR: 104–131) vs. 114% (IQR: 102–126), respectively, *P* = 0.222] (*Figure 2B*). In multivariate analyses, patients of male sex (OR = 2.454, 95% CI: 1.023–5.886, *P* = 0.044), with diabetes (OR = 10–152, 95% CI: 2.581–39.930, *P* = 0.001), and CFR <2.5 in coronary lesions with 30–90% diameter stenosis (OR = 2.279, 95% CI: 1.081–4.807, *P* = 0.030) were associated with haemodynamically obstructive CAD (*Table 3*).

**Table 3:** Impact of demographic variables and invasive measures on the presence of haemodynamically obstructive CAD

	Univariate analysis		Multivariate analysis	
	OR (95% CI)	*P*-value	OR (95% CI)	*P*-value
Age	0.988 (0.961–1.015)	0.381	0.967 (0.921–1.015)^a^	0.176
Male	2.596 (1.552–4.343)	< 0.001	2.454 (1.023–5.886)^a^	0.044
Hypertension	1.171 (0.764–1.795)	0.470	0.727 (0.344–1.536)^a^	0.404
Diabetes	2.111 (1.034–4.310)	0.040	10.152 (2.581–39.930)^a^	0.001
BMI	0.999 (0.946–1.055)	0.975	1.048 (0.949–1.158)^a^	0.355
Active smoking	1.192 (0.739–1.922)	0.471	1.035 (0.455–2.356)^a^	0.934
Median predicted LVM^b^	1.045 (0.987–1.106)	0.134	1.065 (0.960–1.181)^a^	0.236
CFR < 2.5	1.482 (0.789–2.783)^a^	0.221	2.279 (1.081–4.807)^a^	0.030
IMR > 25	0.502 (0.241–1.048)^a^	0.067	0.456 (0.0.195–1.065)^a^	0.070

Univariate and multivariate regression analysis between dependent variable (haemodynamically obstructive CAD) and independent variables (age, sex, hypertension, diabetes, BMI, active smoking, LVM, CFR, and IMR). Data expressed with OR (95% CI).

^a^Analyses performed in patient with coronary lesions with visual DS 30–90% (*n* = 183)

^b^For every 5% increase in median predicted LVM.

CAD, coronary artery disease; OR, odds ratio; CI, confidence interval; BMI, body mass index; LVM, left ventricular mass; CFR, coronary flow reserve; IMR, index of microvascular resistance.

### LVM and invasive haemodynamic parameters based on CMR and ICA

Patient and haemodynamic characteristics across four binary groups based on CMR abnormality and the presence of haemodynamically obstructive CAD are displayed in *Table 4*. In total, 172/354 (49%) patients had concordant normal CMR and no haemodynamically obstructive CAD, 94/354 (27%) patients had concordant abnormal CMR and haemodynamically obstructive CAD, 64/354 (18%) patients had discordant normal CMR and haemodynamically obstructive CAD, and 24/354 (6.8%) patients had discordant abnormal CMR and no haemodynamically obstructive CAD (*Table 4a*).

**Table 4:** Subject and haemodynamic characteristics according to CMR and ICA outcome

	− CMR^a^		+ CMR^b^		*P*-value
	− ICA^a^	+ ICA^b^	− ICA^a^	+ ICA^b^	
**a. Patient characteristics (*n* = 354)**					
*n*	172	64	24	94	
Age [years]	65 (59–70)	65 (57–70)	63 (59–69)	63 (58–69)	0.869
Male	103 (60)	48 (75)	19 (79)	81 (86)	<0.001
Age >65 years	82 (48)	31 (48)	9 (38)	42 (45)	0.779
Hypertension	84 (49)	31 (48)	15 (63)	50 (53)	0.587
Diabetes	7 (4)	14 (22)	3 (13)	10 (11)	0.001
BMI	26 (24–29)	27 (25–29)	28 (26–34)	28 (25–29)	0.097
Active smoking	60 (35)	25 (39)	5 (21)	28 (30)	0.785
Family history of CAD^c^	69 (40)	21 (33)	8 (33)	35 (38)	0.717
Median predicted LVM	111 (101–124)	113 (100–126)	124 (120–137)	118 (108–133)	<0.001
**b. Coronary lesions with visual DS 30–90% (*n* = 183)**					
*n*	98	44	10	31	
FFR (*n* = 158)	0.88 (0.84–0.92)	0.73 (0.68–0.77)	0.86 (0.85–0.93)	0.73 (0.70–0.77)	<0.001
CFR (*n* = 166)	2.6 (1.7–3.4)	2.1 (1.8–2.8)	2.5 (1.7–3.1)	2.6 (1.7–3.0)	0.601
IMR (*n* = 165)	19 (14–29)	19 (16–26)	29 (21–38)	17 (12–22)	0.034

Variables analysed with multinomial logistic regression. Values expressed as *n* (%) or median (IQR). *P*-values were calculated by Kruskal–Wallis *H* test.

^a^The minus symbol (−) represents a normal outcome of CMR or ICA.

^b^The plus symbol represents an abnormal outcome of CMR and/or ICA. An abnormal CMR was defined as a significant perfusion defect in ≥2 contiguous segments and/or LGE in ≥2 contiguous segments and/or wall motion abnormalities in ≥2 contiguous segments. Haemodynamically obstructive CAD was defined as a visual diameter stenosis >90% or FFR ≤0.80 in lesions with 30–90% diameter stenosis during ICA.

^c^Defined as a 1st degree relative with early signs of ischaemic heart disease (men <55 years old, women <65 years old).

CMR, cardiac magnetic resonance; ICA, invasive coronary angiography; BMI, body mass index; CAD, coronary artery disease; LVM, left ventricular mass; DS, diameter stenosis; FFR, fractional flow reserve; CFR, coronary flow reserve; IMR, index of microvascular resistance.

**Figure 3:**
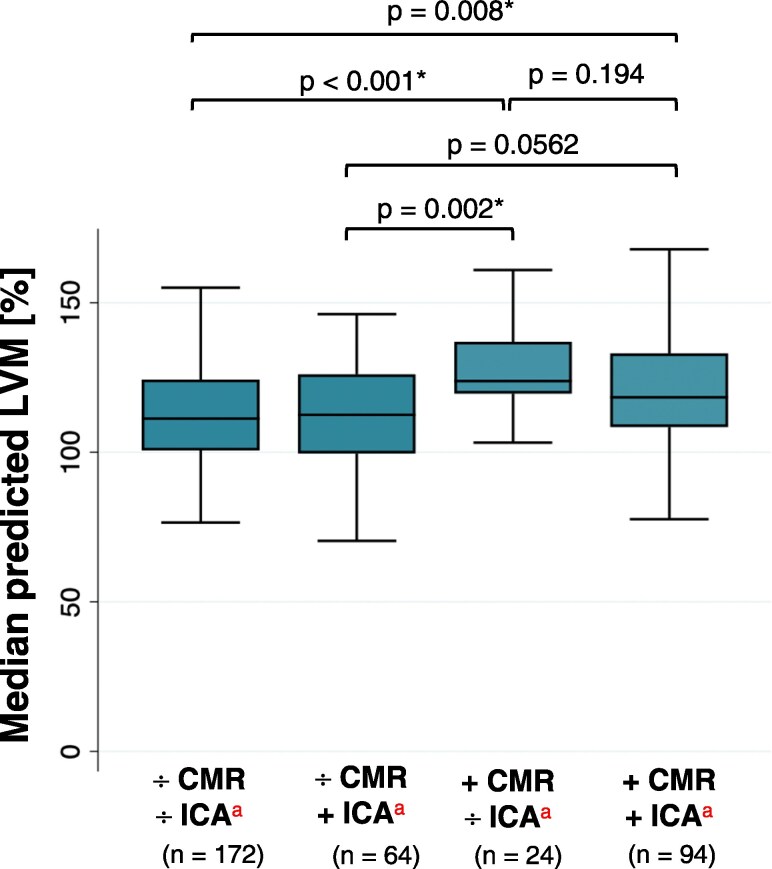
Sex-adjusted median predicted LVM stratified by CMR and ICA. Median (IQR) predicted LVM [%] within binary groups stratified by CMR abnormality and presence of haemodynamically obstructive CAD. Difference between groups was calculated by Dunn’s test. Significant *P*-values marked with *. ^a^The plus symbol (+) represents an abnormal outcome of CMR and/or ICA. The minus symbol (−) represents a normal outcome of CMR and/or ICA. LVM, left ventricular mass; CMR, cardiac magnetic resonance; ICA, invasive coronary angiography.

Patients with discordant abnormal CMR and no haemodynamically obstructive CAD [median predicted LVM = 124% (IQR: 120–137)], and patients with concordant abnormal CMR and haemodynamically obstructive CAD [median predicted LVM = 118% (IQR: 108–133)] had the highest predicted LVM (*Table 4A*), with no difference between the two groups (*P* = 0.194) (*Figure 3*). The greatest difference in LVM was found between the former group and patients with concordant normal CMR and no haemodynamically obstructive CAD (median LVM difference = 13%, *P* = 0.001). Patients with an abnormal CMR and haemodynamically obstructive CAD had higher LVM than patients with normal CMR and no haemodynamically obstructive CAD (median LVM difference = 7.0%, *P* = 0.008) (*Figure 3*).

**Figure 4:**
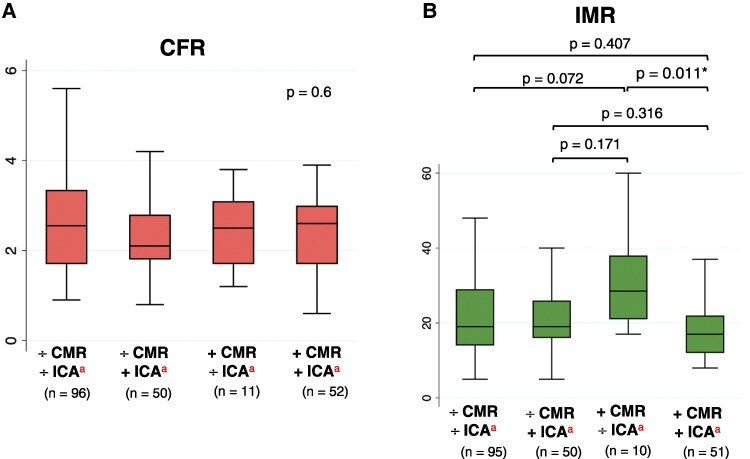
Box plots of CFR and IMR stratified by CMR and ICA. Boxplots displaying median (IQR) *A*) CFR and *B*) IMR in coronary lesions with DS 30–90% within binary groups stratified by CMR abnormality and by presence of haemodynamically obstructive CAD. Difference between groups was calculated using Dunn’s test. Significant *P*-values marked with *. *P*-values for intergroup for CFR are not calculated because the Kruskal–Wallis *H* test was non-significant (*P* = 0.6). ^a^The plus symbol (+) represents an abnormal outcome of CMR and/or ICA. The minus symbol (−) represents a normal outcome of CMR and/or ICA. CFR, coronary flow reserve; IMR, index of microvascular resistance; CMR, cardiac magnetic resonance; ICA, invasive coronary angiography.

Across groups, CFR was only below the pre-defined abnormality threshold in patients with discordant normal CMR and haemodynamically obstructive CAD [median CFR = 2.1 (IQR: 1.8–2.8)], and no difference in median CFR levels was found between the groups (Kruskal–Wallis *H* test = 0.601) (*Table 4B*) (*Figure 4A*). IMR was highest in patients with discordant abnormal CMR and no haemodynamically obstructive CAD [median IMR = 29 (IQR: 21–38)] and was higher compared to patients with concordant abnormal CMR and haemodynamically obstructive CAD (median IMR difference = 12, *P* = 0.011) (*Figure 4B*).

## Discussion

This multicentre study aimed to assess the impact of LVM on discordant findings between CMR imaging and invasive coronary angiography (ICA) in patients with suspected stenosis on CCTA. Regardless of the presence of haemodynamically obstructive CAD, sex-adjusted LVM was higher in patients with abnormal CMR results. Patients with abnormal CMR findings but no haemodynamically obstructive disease on ICA had both elevated LVM and increased IMR. These findings suggest that CMR results may be falsely positive due to LVH, or that ICA with fractional flow reserve (FFR) may yield false negatives due to high IMR. Therefore, assessment of LVM may be an important factor to consider when evaluating patients with new-onset chest pain and clinically suspected obstructive CAD using both CMR and FFR.

### The clinical application of CMR

The performance of CMR to assess CAD has been extensively investigated. Several studies report high sensitivities and specificities in cohorts with high-risk patients and high disease prevalence using haemodynamically obstructive CAD as a reference standard.^12–16^ However, in patients with moderate stenoses yielding positive FFR values, rather low sensitivities have been demonstrated.^6,7,17^ This is in alignment with our findings, where concordant abnormal CMR and ICA findings are present in 94/354 (27%) patients. Compared to previous studies, besides inclusion of patients with known CAD,^13–15^ yielding higher disease burdens^12,18,19^ and dissimilar definitions of CMR abnormality,^16^ reasons for the rather high rate of discordant CMR and ICA results could be partly attributable to LVM, which was higher in patients with discordant abnormal stress CMR and no haemodynamically obstructive CAD (*Table 4a*).

### Determinants of increased LVM and its diagnostic yield

A longitudinal evaluation of LVM in the Framingham cohort revealed that an abnormal left ventricular (LV) geometry and LVH are associated with an increased risk of cardiovascular events.^20^ The most significant risk factors influencing LVM are systemic blood pressure, male sex, age, and BMI. Patients in the two groups with the highest LVM i.e. patients with an abnormal CMR with and without haemodynamically obstructive CAD, were overweight, male patients with a high prevalence of hypertension (*Table 4a*). This was also consistent with the LVM index (LVMI) being increased but below the hypertrophic threshold when compared to patients with a normal CMR. A linear regression between LVM and BMI indicated that increasing BMI reduced LVM (see Supplementary data online, *Figure S1*). However, in a large cohort study, obesity (BMI ≥30 kg/m^2^) was associated with increased LVM.^21^ The inconsistent results can be explained by patients in this study being overweight (median BMI = 27 kg/m^2^), and not obese. Within the two groups, LVM was highest in patients with discordant abnormal CMR and no haemodynamically obstructive CAD. This could further suggest that increased LVM detected on CMR may contribute as an independent indicator of myocardial ischaemia regardless of epicardial dysfunction at ICA, which warrants further investigation (*Table 4a*).

### The impact of LVM on microvascular function

In this study, patients with the highest LVM, namely those with discordant abnormal CMR and no haemodynamically obstructive CAD, presented with elevated IMR and normal FFR. This is consistent with coronary blood flow increasing proportionally with LVM, but the relative flow to mass ratio decreases.^22^ Given the inverse relationship between flow and resistance according to Ohm’s law of resistance, increased LVM leads to increased microvascular resistance relative to LVM, and accordingly, the pressure gradient across an epicardial stenosis decreases, resulting in a higher FFR.^23^ Myocardial remodelling following LVM enlargement is known to predispose to isolated coronary microvascular disease, a known cause of myocardial infarction with non-obstructive coronary arteries (MINOCA).^24^ While the clinical presentation of MINOCA can be indistinguishable from obstructive myocardial infarction, invasive angiographic findings include non-obstructive CAD and increased IMR. Thus, microvascular dysfunction, with or without epicardial stenosis, could cause a scenario of combined normal FFR, elevated IMR, and low coronary blood flow. This may explain patients presenting with discordant normal FFR and abnormal CMR in this study.

Conversely, the opposite scenario entails impaired FFR and normal CMR, indicating high coronary blood flow and low microvascular resistance. As expected, patients with concordant normal CMR and ICA findings had preserved FFR and low IMR, suggesting no significant CAD. Patients with impaired FFR also presented with lower IMR. A common feature was lower LVM compared with those having discordant abnormal CMR but normal ICA. This corresponds to the coherent relationship between epicardial and microvascular physiology, where a decrease in epicardial pressure reduces the microvascular perfusion.

In both scenarios, CMR would appear either less sensitive or specific for obstructive CAD if FFR is used as gold standard. This could be due to dissimilar definitions of obstructive CAD by CMR and FFR, and their diagnostic yield. Moreover, CMR primarily provides measures of anatomy, volume, and perfusion on a myocardial level to detect ischaemia, while FFR is used to assess the functional severity of coronary lesions and, consequently, to guide revascularization.^1^ Thus, patients may have myocardial perfusion defects without obstructive CAD. For this reason, suspected obstructive CAD detected on CMR may contribute as a second-line non-invasive tool to not only refer selective patients to ICA with FFR measurements but also to consider MINOCA.

### Limitations

This study has some limitations. First, primarily low-intermediate-risk patients were included, which may have affected the prevalence and detection rate of CAD by CMR compared to an all-comer population. Therefore, the external validity of the findings is limited by the inclusion of only new onset stable chest pain patients with suspected obstructive disease on CCTA.

Visual assessment of diameter stenosis on ICA may influence the internal consistency, resulting in operator-specific outcomes. Invasive physiological measurements during ICA were assessed in coronary lesions with a visual diameter stenosis of 30–90%. If diameter stenoses were under- or overestimated by visual assessment (<30% or >90%), more vessels would be non-suitable for physiological measures. The guideline from the European Society of Cardiology recommends invasive functional testing during ICA in vessels with a diameter stenosis of 40–90%. This means that FFR in this study was potentially assessed in vessels with non-significant lesions, resulting in a low sensitivity of ICA with visual assessment and FFR as reference.

LVM was not estimated according to vessel-specific territories, and patients were not stratified by the number of diseased vessels. This constitutes a discrepancy in the use of a global vs. a vessel-specific LVM, with assessment of LVM based on ischaemic vessel territories being more specific.

## Conclusion

In patients with stable chest pain and suspected obstructive CAD on CCTA, increased LVM can potentially confound concordance between stress CMR and ICA. This is due to increased microvascular resistance, which decreases the pressure gradient across an epicardial stenosis, resulting in an apparent high FFR and thus classified as a normal ICA.

## Supplementary Material

jeaf350_Supplementary_Data

## Data Availability

No new data were generated or analysed in support of this research.
