# Supplementary material for: Association of left ventricular mass with discordant stress cardiac magnetic resonance and coronary angiography
Source: Eur Heart J Cardiovasc Imaging. 2026 Feb 10;27(4):629–37. doi: 10.1093/ehjci/jeaf350 (PMC13021273; doi:10.1093/ehjci/jeaf350)
Supplement: jeaf350_Supplementary_Data [file jeaf350_supplementary_data.docx]

**SUPPLEMENTAL DATA**

# Supplemental equation 1. Sex-adjustment of LVM

LVM was sex-adjusted from grams to percent using following formula:

$$100\%*LVM\left( a \times height^{0.54} \times weight^{0.61} \right)$$

where a=8.25 for males and a=6.82 for females.

**Supplemental Figure 1.**


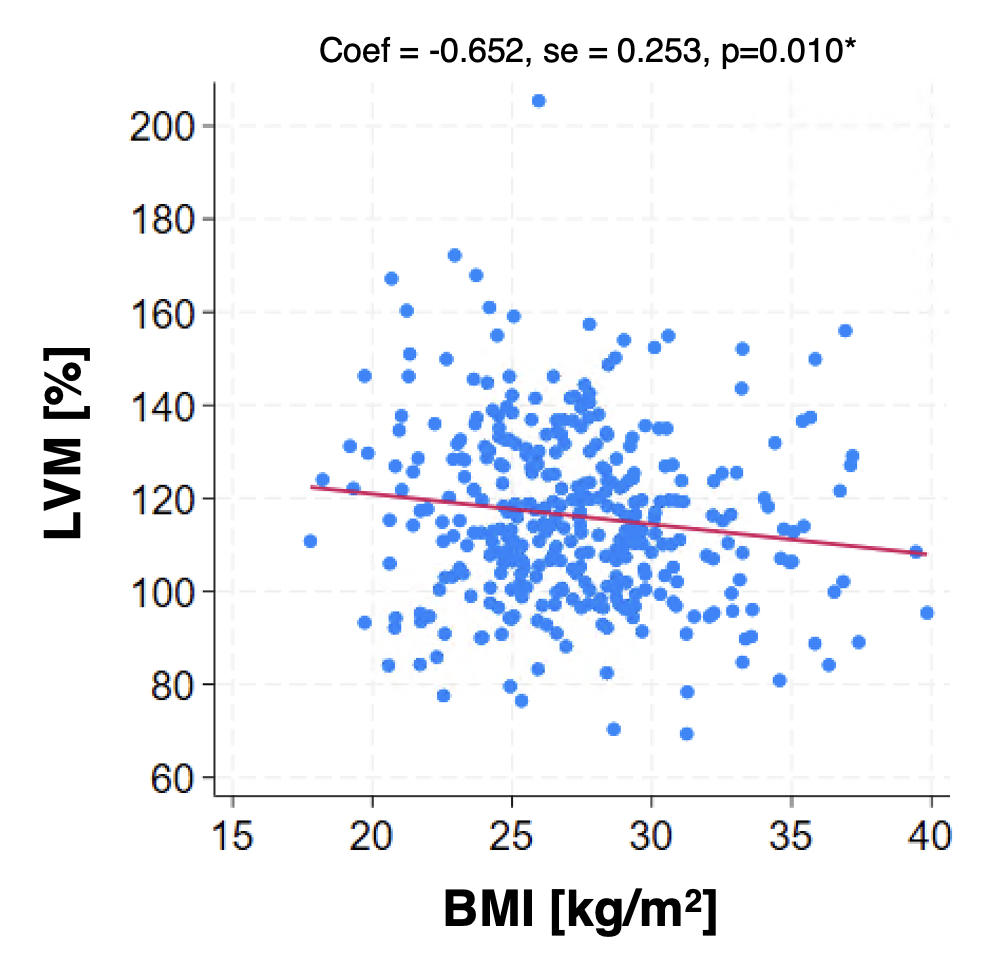


Two-way scatter plot showing correlation between LVM and BMI with display of coefficient (coef), standard error (se), and p-value.

* Significant p-value

LVM = left ventricular mass. BMI = body mass index.
